# Proceedings of the Mississppi Valley Association of Dental Surgeons

**Published:** 1852-10

**Authors:** 


### DENTAL REGISTER OF THE WEST.

VOL. VI. OCTOBER, 1852, No. 1.

### Original Communications.

### THE PROCEEDINGS,

*Of the Mississipi Valley Association of Dental Surgeons.*

*Cincinnati, Sept. 8th.*

The eighth annual meeting of the Association commenced its sittings in the Ohio College of Dental Surgery, at 10 A. M. of Wednesday, Sept. 18th, 1852. Dr. Goddard, 3d vice president in the chair.

On calling the roll, the following members answered to their names, viz:

Drs. Goddard, M. Cullum, Bonsall, Baxter, and A. M. Hunt.

On motion of Dr. Hunt, Dr. Bonsall was appointed secretary pro tem. Neither of the executive committee being present, on motion, Drs. Hunt and Baxter, were appointed a committee to prepare business—on motion of Dr. Bonsall, adjourned to meet at 10 A. M. to-morrow.

THURSDAY MORNING, SEPTEMBER, 9th.

The society met according to adjournment, Dr. Ulrey, 1st vice president in the chair, in addition to the members present yesterday, Drs. James Taylor, J. P. Ulrey, Van Emon, Leslie and Taft, appeared and took their seats; Drs. Watt and Hamill were present by invitation. The minutes of yesterday were read and adopted, and then the following report was received from the committee on business.

*To the Mississippi Valley Association of Dental Surgeons.*

The committee appointed to prepare and arrange business for the action of the society, respectfully report that they recommend proceedings as follows:

1st. Report of the examining committee.

2d. Reports of officers of the Association.

3d. Reports of standing and special committees.

4th. Addresses by gentlemen appointed for that purpose at the last annual meeting.

5th. Consideration of any subjects which may be deemed of peculiar importance in relation to operative or mechanical dentistry.

6th. Your committee would also respectfully submit to the Association, that some well defined action be taken in respect to dental patents, as your committee believe that the profession abroad, and the public generally, labor under a mistaken impression of the views of the members of this Association on that subject, and your committee would offer for adoption the following resolution.

*Resolved*, That a committee of three, be appointed to present such resolutions on the subject of dental patents as they may think advisable, and that said report be made the order of the day, at 2 P. M. this day.

Signed, A. M. Hunt.

J. W. Baxter.

The report of the committee on business was adopted, and the chairman appointed Drs. Taft, Leslie and Van Emon, as the committee on patents.

On motion of Dr. Taylor, Drs. Hunt and Taft were appointed an auditing committee to report this afternoon—the examining committee now reported Drs. Geo. Watt of Xenia,

Ohio, and John G. Hamill, of Ripley, as competent dentists, and worthy to become members of the Asssociation, and they were unanimously elected—the balance of the morning was devoted to the discussion of various subjects of dental interest. On motion adjourned till 2 P. M.

---

AFTERNOON SESSION.

The society met at 2 P. M. and the majority of the committee reported as follows.

The committee appointed on the subject of patenting improvements in dentistry respectfully report: That inasmuch as our profession is a department of a *liberal* profession and inasmuch as the wisest, most talented and liberal among us, cherish most cordially the free interchange of ideas and modes of practice; your committee recommend the adoption of the following resolution.

*Resolved*, That it is now and always has been the sentiment of this society, that it is derogatory to the professional character of any of its members, to patent any dental instrument or mode of practice.

Signed, J. TAFT.
A. M. LESLIE,

Dr. Van Emon, the minority of the committee had not prepared any report, but stated verbally that he could not concur fully in the report. The reports were accepted, and after considerable discussion by Drs. Leslie, Goddard, Taylor, Van Emon, Hunt, Taft and others, the report was adopted.

Dr. Taylor as one of the committe, on Dr. Drake's enquiries reported progress, and they were directed to send their report when finished to Dr. Drake for publication, and also to publish it in the Register, as the report of the committee.

The auditing committee now reported as follows, viz. that they have examined the books of the editor of the Register and find the receipts for the last year, to be,—

| | |
|---|---|
| For subscription. - - - - - - | $206.00 |
| Advertising, - . - - - - - | 12.00 |
| | $218.00 |
| Cost of publication, - - - - | 246.00 |
| Leaving a deficiency, - - - - - | $28.00 |

They also reported having examined the Treasurer's accounts and found a balance in his hands of forty-five dollars and twenty-six cents. After the adoption of the report, it was on motion of Dr. Ulrey,

*Resolved*, That we tender to Dr. James Taylor, the back numbers of the Dental Register and the books, &c., received in exchange, also the subscriptions, now due for it, as a remuneration for his labor, and that he be requested to pay the debts due by the Register, and continue the publication on his own responsibility. Dr. Taylor signified his consent to this arrangement.

Dr. Taft's address being next in order, was read by him, and he was requested to continue the subject at the next meeting.

On motion, adjourned to meet at the office of Dr. Taylor, at 7½ P. M.

---

EVENING SESSION.

The society met according to adjournment, and after the adoption of the previous minutes, it was on motion, of Dr. Goddard,

*Resolved*, That this Association invite its members to present at its next annual meeting all discoveries and improvements which they may have recently made in dentistry, and that this Association will award a gold medal to him who shall produce and demonstrate the most valuable improvement or invention, the same to be decided by a majority vote of the members present.

On motion of Dr. Baxter, it was,

*Resolved,* That the Association will hereafter, after the payment of its necessary expenses, devote the most of the balance of its funds to the formation of a fund, for the purpose of rewards for valuable improvements appertaining to the profession of dentistry, to be decided on at each annual meeting.

On motion of Dr. Taylor,

*Resolved,* That the Association award a medal worth twenty dollars, on the best set of extracting instruments exhibited at our next annual meeting.

A communication was received from Dr. Van Emon, resigning his membership, which the society agreed to accept, when his dues were all paid up.

On motion of Dr. Goddard, it was unanimously decided to amend the constitution, so as to have but one Vice President hereafter.

The society now went into an election for officers, when the following were elected.

Dr. J. Taft, *President.*

Dr. A. M. Hunt, *Vice President.*

Dr. A. M. Leslie, *Recording Secretary.*

Dr. W. H. Goddard, *Corresponding Secretary.*

Dr. C. Bonsall, *Treasurer.*

Drs. Berry, Goddard and McCullum, *Examining Committee.*

Drs. Ulrey, Watt and Baxter, *Executive Committee.*

On motion of Dr. Bonsall, it was,

*Resolved,* That when we adjourn, we do so, to meet on the day following, or preceding the annual examination of the Ohio Dental College.

On motion the treasurer was directed to pay to the janitor of the college, $2 for services rendered.

Drs. Watt, A. M. Hunt and Hamill, were appointed to deliver addresses at the next meeting. Dr. Leslie, now exhibited several specimens of Dr. Hunters new style of teeth, with receipts for making teeth, body, enamel, &c. for which a vote of thanks was ordered to Drs. Hunter and Leslie.

On motion, adjourned, to meet in the Ohio Dental College, at 10 A. M. of Wednesday, 17th February next.

CHAS. BONSALL, *Secretary, pro tem.*

